# Pharmacological Modulation of Long-Term Potentiation-Like Activity in the Dorsolateral Prefrontal Cortex

**DOI:** 10.3389/fnhum.2018.00155

**Published:** 2018-04-24

**Authors:** Bahar Salavati, Zafiris J. Daskalakis, Reza Zomorrodi, Daniel M. Blumberger, Robert Chen, Bruce G. Pollock, Tarek K. Rajji

**Affiliations:** ^1^Geriatric Psychiatry Division, Centre for Addiction and Mental Health, Toronto, ON, Canada; ^2^Department of Psychiatry, University of Toronto, Toronto, ON, Canada; ^3^Campbell Family Mental Health Research Institute, Centre for Addiction and Mental Health, Toronto, ON, Canada; ^4^Krembil Research Institute, University Health Network, Toronto, ON, Canada

**Keywords:** electroencephalography, neuroplasticity, paired associative stimulation, pharmacology, transcranial magnetic stimulation

## Abstract

**Background**: Long-term potentiation (LTP) depends on glutamatergic neurotransmission and is modulated by cholinergic, dopaminergic and GABAergic inputs. Paired associative stimulation (PAS) is a neurostimulation paradigm that, when combined with electroencephalography (EEG), assesses LTP-like activity (PAS-induced LTP) in the dorsolateral prefrontal cortex (DLPFC). Thus, we conducted a study to assess the role of cholinergic, dopaminergic, GABAergic and glutamatergic neurotransmission on PAS-induced LTP in the DLPFC. We hypothesized that increasing the dopaminergic tone with L-DOPA and the cholinergic tone with rivastigmine will enhance PAS-induced LTP, while increasing the GABAergic tone with baclofen and inhibiting glutamatergic neurotransmission with dextromethorphan will reduce it compared to placebo.

**Methods**: In this randomized controlled, double-blind cross-over within-subject study, 12 healthy participants received five sessions of PAS to the DLPFC in a random order, each preceded by the administration of placebo or one of the four active drugs. PAS-induced LTP was assessed after each drug administration and compared to PAS-induced LTP after placebo.

**Results**: As predicted, L-DOPA and rivastigmine resulted in enhanced PAS-induced LTP in the DLPFC and dextromethorphan inhibited it compared to placebo. In contrast, baclofen did not significantly suppress PAS-induced LTP compared to placebo.

**Conclusions**: This study provides a novel approach to study DLPFC neuroplasticity and its modulation in patients with brain disorders that are associated with abnormalities in these neurochemical systems. This study was based on a single dose administration of each drug. Given that these drugs are typically administered chronically, future studies should assess the effects of chronic administration.

## Introduction

Neuroplasticity refers to the ability of the brain to change and adapt in response to experiences (Pascual-Leone et al., 2005). Long-term potentiation (LTP) is a synaptic form of neuroplasticity that is considered to be fundamental for learning and memory (Collingridge and Bliss, 1995). The dorsolateral prefrontal cortex (DLPFC) plays an important role in several cognitive functions including learning and memory (Fuster, 2008). Further, abnormalities in the DLPFC structure and function are observed in various brain disorders including Alzheimer’s disease (Kaufman et al., 2010), depression (Koenigs and Grafman, 2009) and schizophrenia (Callicott et al., 2000). Thus, studying LTP and its modulation in the DLPFC could advance knowledge of DLPFC function and lead to the development of effective cognitive interventions for these brain disorders.

Paired associative stimulation (PAS) is a neurostimulation paradigm that induces *in vivo* LTP-like activity in the human cortex (Stefan et al., 2000; Rajji et al., 2013). PAS simulates a spike-timing dependent plasticity protocol by combining single-pulse transcranial magnetic stimulation (TMS) to a cortical area with contralateral peripheral nerve stimulation (PNS) such as the two stimulations arrive contemporaneously at the targeted cortical area and in turn strengthen the cortical output in response to single-pulse TMS. Using well-established methods of combining TMS with electroencephalography (EEG), our group has shown that PAS results in LTP-like activity in the human DLPFC as captured by EEG as the potentiation of TMS-induced cortical evoked activity (CEA; Rajji et al., 2013; Kumar et al., 2017; Loheswaran et al., 2017). This PAS-induced LTP-like activity only simulates cellular LTP and will be referred to hereafter as PAS-induced LTP merely for simplicity. PAS-induced LTP has also been shown to be impaired in several brain disorders, e.g., Alzheimer’s disease (Battaglia et al., 2007; Kumar et al., 2017), depression (Player et al., 2013) and schizophrenia (Frantseva et al., 2008).

The pathway that is thought to be targeted by applying PAS to the DLPFC is a pathway that connects the somatosensory cortex to the prefrontal cortex as supported by neuroanatomical and neurophysiological studies in rodents (Van Eden et al., 1992; Monconduit et al., 1999; Golmayo et al., 2003) and non-human primates (Petrides and Pandya, 1984; Goldman-Rakic, 1988). Human neurophysiological studies also support this pathway as stimulation of the median nerve results in an evoked potential over the contralateral prefrontal cortex (García Larrea et al., 1992; Valeriani et al., 1997, 1998).

Synaptic LTP depends on glutamatergic neurotransmission (Lüscher and Malenka, 2012) and is modulated by cholinergic (Picciotto et al., 2012), dopaminergic (Tritsch and Sabatini, 2012) and GABA-ergic (Nugent and Kauer, 2008) neurotransmission. A few studies have assessed the pharmacological modulation of PAS in the human motor cortex with output response measured indirectly from motor evoked potentials (MEPs) not cortically using EEG. Previously, it has been shown that baclofen (50 mg), which increases GABA-ergic tone, decreases PAS-induced LTP (McDonnell et al., 2007). In contrast, dextromethorphan (150 mg), which has been shown to block NMDA glutamatergic receptors, decreases PAS-induced LTP (Stefan et al., 2002; Weise et al., 2017). Further, L-DOPA (100 mg), which increases dopaminergic tone, increases LTP (Thirugnanasambandam et al., 2011). Lastly, rivastigmine (3 mg), which increases cholinergic tone, enhances PAS-induced LTP (Kuo et al., 2007).

Despite the important role the DLPFC plays in cognition, to date, no study has assessed the pharmacological modulation of PAS-induced LTP in this bran region. Further, no study has assessed all of these drugs in the same participants and not all of the above studies conducted in the motor cortex were double-blind or randomized. Also, no study to date has analyzed the pharmacological effects of PAS-LTP using EEG. Thus, using a double-blind randomized controlled within-subject design that included all of the above four drugs we conducted the first pharmacological modulation study of DLPFC plasticity *in vivo* using PAS-EEG. We hypothesized that, compared to placebo, L-DOPA and rivastigmine would increase PAS-induced LTP, while baclofen would decrease it and dextromethorphan would block it.

## Materials and Methods

### Experimental Design

The current study was a double-blind randomized controlled within-subject cross-overdesign. It consisted of five study visits where each participant received five sessions of PAS to the DLPFC in a random order, each followed by the administration of placebo or one of the four active drugs, and separated by at least 1 week to minimize drug interference and carryover effects. The time for each drug administration before PAS was based on the time of the drugs plasma peak, i.e., 1 h for baclofen, 3 h for dextromethorphan, 1 h for L-DOPA and 2 h for rivastigmine. The placebo was randomly given to each participant at 1, 2 or 3 h prior to the administration of PAS. The doses of the drugs (Baclofen 50 mg, dextromethorphan 150 mg, L-DOPA 100 mg and rivastigmine 3 mg) were based on the previous studies demonstrating effects at similar doses on PAS-induced LTP in the motor cortex (Stefan et al., 2002; Kuo et al., 2007; McDonnell et al., 2007; Thirugnanasambandam et al., 2011). Across the participants, the sequences of drug administration were counterbalanced. The administrator of the experiments and participants were blind to drug assignment. All data processing and analyses were also completed under blind condition.

### Participants

Participants were females and males; aged 18–55 years because cortical neuroplasticity as measured using neurophysiologic methods starts to decline around age 50 (Müller-Dahlhaus et al., 2008); healthy not diagnosed with any neurologic or psychiatric disorder (all participants were drug tested); non-smokers; right-handed to ensure homogeneity in hemisphere dominance; had no contraindication to TMS (Rossi et al., 2009) or MRI; and provided written informed consent. Also, pregnancy was ruled out by a urine test in female participants. The protocol was approved by the Centre for Addiction and Mental Health Research Ethics Board. All subjects gave written informed consent before participation.

### Locating and Co-registering the DLPFC

Each participant’s T1-weighted MRI with fiducial markers placed on the nasion, inion, left and right tragus and vertex was used to locate the left DLPFC (Rajji et al., 2013; Sun et al., 2016). The left DLPFC is located at the junction of the middle and anterior third of the middle frontal gyrus (Talairach Co-ordinates (x, y, z) = (−50, 30, 36), which corresponds to the posterior region of Brodmann area 9 and the superior section of area 46. The localization of the DLPFC was achieved through neuronavigation techniques using the MINIBIRD system (Ascension Technologies, Shelburne, VT, USA). The MRI acquisition parameters were the following: GE Discovery MR 750, 3 Tesla, Repetition Time = 7 ms, Echo Time = 3 ms, Flip Angle = 8°, Slice Thickness = 0.9 mm, Number of Slices = 128, Voxel Size = 0.9 × 0.9 × 0.9 mm.

### Electromyography (EMG) Recordings From the Motor Cortex and TMS-EEG in the DLPFC

**Figure 1:**
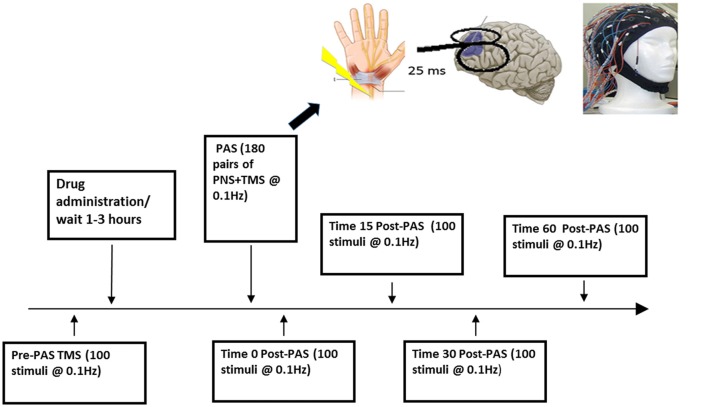
Experimental design. This figure illustrates one session of the paired associative stimulation (PAS) protocol.

At the beginning of each study, we used a 7 cm figure-eight coil and a Magstim 200 stimulator (The Magstim Company, Whitland, UK) to determine the participant’s resting motor threshold (RMT; defined as the minimum stimulus intensity that elicits a MEP of more than 50 μV in 5 of 10 trials) from stimulating the left motor cortex at the optimal location for obtaining an MEP from right abductor pollicis brevis muscle. MEP activity was measured through electromyography (EMG) recordings from the abductor pollicis brevis. This signal was recorded by placing two disposable electrodes over the right abductor pollicis brevis, which was then amplified using a Model 2024 amplifier and was filtered at a band pass of 2–2.5 Hz and digitized using micro1401 (Cambridge Electronics Design, Cambridge UK; Rajji et al., 2013; Sun et al., 2016). The RMT was then adjusted to a suprathreshold intensity to produce a mean peak-to-peak MEP amplitude of ~1 mV over 20 trials, which corresponded to approximately 120% of the RMT (Rajji et al., 2013). This intensity referred to as SI 1 mV was then used to deliver 100 single TMS pulses at 0.1 Hz to the scalp over the left DLPFC during pre-PAS while EEG was being recorded to determine baseline CEA then again for post-PAS 0, 17, 34 and 60 min to assess change in CEA (Figure 1; Rajji et al., 2013; Loheswaran et al., 2017). The left DLPFC determined by the participant’s MRI image was marked on the EEG cap with a marker to ensure identical placement throughout the experiment. When stimulating the left DLPFC the handle of the TMS coil was pointed backwards, at approximately 45° to the midsagittal line.

During pre-PAS and post-PAS CEA was acquired through a 64-channel Synamps 2 (Neuroscan Inc., Charlotte, NC, USA) EEG system. All electrodes (Ag/AgCl ring electrodes) impedance were ≤5 kΩ and referenced to an electrode positioned posterior to Cz electrode. In addition, EEG signals were recorded using DC and a low-pass anti-aliasing filter, of 200 Hz, at 20 kHz sampling rate, which has been shown to avoid saturation of amplifiers and minimize TMS-related artifact (Rajji et al., 2013; Loheswaran et al., 2017).

### PAS to the DLFPC

PAS was administered to the DLPFC and consisted of 180 simultaneous paired pulses of PNS to the median nerve followed 25 ms later by a TMS pulse to the scalp over the left DLPFC during a 30 min period at 0.1 Hz. This paradigm has been shown to induce LTP-like activity by potentiating CEA over the DLPFC in healthy individuals (Rajji et al., 2013; Loheswaran et al., 2017). PNS was delivered at 300% of the sensory threshold, defined as the minimum intensity that the participant perceives sensation. Given that attention affects the level of PAS induced potentiation PAS (Stefan et al., 2004), participants were asked to maintain attention by attending to their wrist and continuously count and randomly report the number of PAS pulses delivered within the 30 min period and at the end of PAS to report their final count (Rajji et al., 2013; Loheswaran et al., 2017).

The disadvantage of giving the drug after pre-PAS rather than before pre-PAS is that the effect post-PAS could be argued is due to a direct effect on CEA and not necessarily through PAS. However, if the drug is given before pre-PAS and its delivery is timed such as its peak corresponds to pre-PAS CEA rather than PAS, then the post-PAS effects may not also be related to PAS but could be due simply to the drug effects on pre-PAS CEA. Further, if two sessions of pre-PAS CEA measurements were administered and the drug was given between these two sessions to assess its impact on CEA before PAS, the effect on PAS *per se* could still be confounded by the fact that the post-PAS effect could be only due to the effect of the drug on the second pre-PAS CEA measurement. In addition the experiment would be become too burdensome on participants and the effects of two pre-PAS sessions on PAS effects are also not known. Finally, several studies showed no significant impact of these drugs on basic neurophysiological measures, e.g., RMT, active motor threshold, or baseline MEP (Ziemann et al., 1998; McDonnell et al., 2006; Kuo et al., 2007; Thirugnanasambandam et al., 2011). Thus, we elected to give the drug after pre-PAS CEA measurement and time the drug such as the peak corresponds to PAS and not post-PAS CEA measurements.

### EEG Data Processing

All analyses was done while blinded and we were only unblinded once the data was finalized. EEG data was analyzed using MATLAB (The MathWorks Inc., Natick, MA, USA) and a custom script that was developed based on previous work (Rajji et al., 2013; Sun et al., 2016; Kumar et al., 2017; Loheswaran et al., 2017; Salavati et al., 2018). First, raw EEG recordings were down sampled from 20 kHz to 1 kHz and then segmented into epochs from −1000 ms to + 2000 ms relative to the onset of the TMS pulse. Each trial was then baseline corrected with the mean of the TMS artifact-free time period (−500 to −110 ms) before the TMS pulse. To minimize TMS artifacts, the data was re-segmented from 25 ms before the TMS pulse to 2000 ms after the TMS pulse. Next, the EEG data was digitally filtered using a second-order, Butterworth, zero-phase shift 1–55 Hz band pass filter (24 dB/Oct). EEG recordings from all five time points of the study (pre and at time 0, 17, 34 and 60 min post-PAS) were then concatenated in order to apply the same objective criteria for cleaning the data. Then, an electrodes-by-trials matrix of ones and zeros was created and assigned a value of zero if an epoch had the following: (1) amplitude larger than ±150 μV; (2) power spectrum that violated 1/f power law; or (3) standard deviation (SD) three times larger than the average of all trials. The power spectral density of EEG signal is inversely proportional to the frequency of the signal, except the alpha band, which shows a stronger power spectrum than 1/f shape (Luck, 2014). The epoch was marked for the final rejection assessment if fitting curve (i.e., nonlinear least squares) to the power spectrum showed R-squared <0.6. An electrode was rejected if its corresponding row had more than 60% of columns (trials) coded as zeros. An epoch was removed if its corresponding column had more than 20% of rows (electrodes) coded as zeros. Next, independent component analysis (ICA; EEGLAB toolbox; Infomax algorithm) was performed to remove remaining artifacts such as eye blink traces, muscle artifacts from the EEG data. Finally, the data was re-referenced to the average, generating a clean signal devoid of noise for each participant.

To determine potentiation of CEA by PAS we first calculated the average of the TMS evoked potential (TEP) at pre-PAS and post-PAS (0, 17, 34, 60 min), from all epochs that corresponded to the electrode at the site of stimulation, i.e., the left DLPFC which was determined by each participant’s MRI image (Rajji et al., 2013; Loheswaran et al., 2017). Then using the Hilbert transform the instantaneous amplitude of TEP signal was extracted. Hilbert transform provided an envelope waveform for TEP signal, which gives a more reliable power estimation for the signal (Freeman, 2007). The area under the rectified CEA curve between 50 and 275 ms post-TMS pulse was then calculated for pre-PAS and post-PAS (0, 17, 34, 60 min). The first interval cut-off (i.e., 50 ms) was chosen as it represents the earliest TMS artifact-free data, while the second interval cut-off (i.e., 275 ms) was chosen because it represents the end of the window during which potentiation of post-PAS CEA is still significant (Rajji et al., 2013; Loheswaran et al., 2017). An example of raw data TEPs is shown in Figure 4.

**Figure 2:**
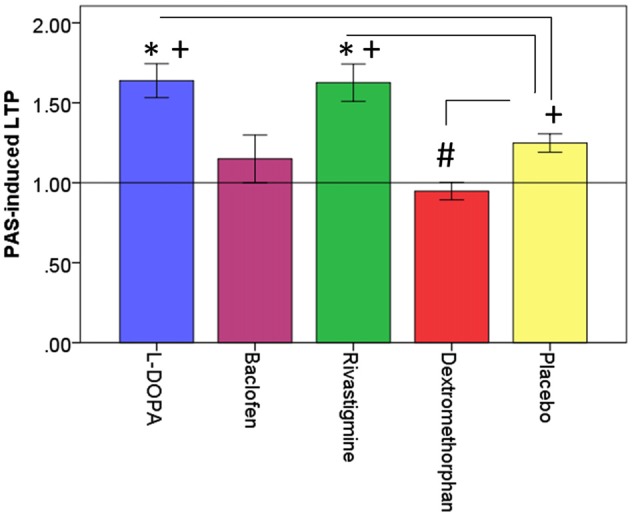
Effects of drugs on dorsolateral prefrontal cortex (DLPFC) neuroplasticity. This figure illustrates the effects of drugs (L-DOPA, baclofen, rivastigmine, dextromethorphan and placebo on PAS-induced long-term potentiation (LTP)-like activity (PAS-induced LTP) expressed as a ratio of post-PAS cortical evoked activity (CEA)/pre-PAS CEA over the DLPFC. *Refers to significant increase in PAS-induced LTP compared to placebo (L-DOPA: *p =* 0.004; Rivastigmine: *p* = 0.009); ^#^refers to significant decrease in PAS-induced LTP compared to placebo (Dextromethorphan: *p* = 0.007). ^+^refers to significant PAS-induced LTP compared to a value of 1 which is represented by the horizontal black line. Error bars: ±1 SE.

**Figure 3:**
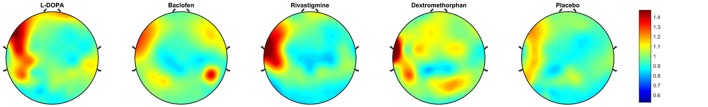
Topoplots of plasticity. These topoplots illustrate the effects of drugs (L-DOPA, baclofen, rivastigmine, dextromethorphan and placebo) on PAS-induced LTP-like activity in the DLPFC.

**Figure 4:**
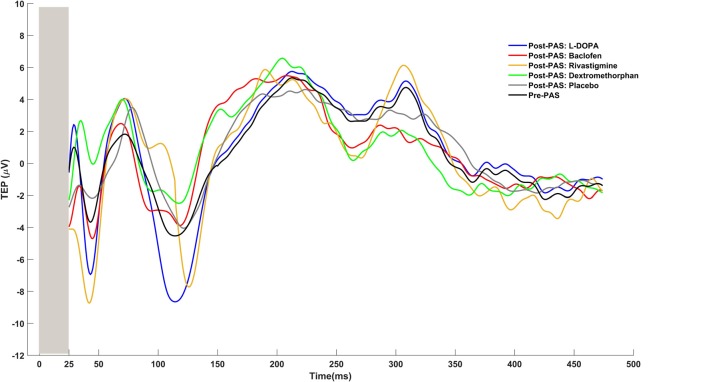
Examples of TMS evoked potentials (TEPs) for pre-PAS and post-PAS under each drug condition.

### Data Analysis

To measure PAS-induced LTP over the DLPFC, we calculated CEA at each time point post-PAS (0, 17, 34 and 60 min) and divided by CEA pre-PAS. The ratio represents potentiation of CEA at each of the time points (0, 17, 34, 60 min) post-PAS (Rajji et al., 2013; Loheswaran et al., 2017). Since the timing of maximum potentiation post-PAS could vary among participants, the maximum CEA ratio among these time points (0, 17, 34, 60 post-PAS) were selected for each participant per condition (Rajji et al., 2013; Loheswaran et al., 2017). This maximum CEA ratio for each drug condition represents PAS-induced LTP for each participant under the influence of that drug. To determine PAS-induced LTP in the DLPFC we used the electrode corresponding to the participant’s DLPFC for two main reasons. First, this is the area of interest and stimulation, and second, previous work has shown that PAS to the DLPFC is focal and localized to the left frontal brain region and greatest in the electrodes overlying the DLPFC (Rajji et al., 2013; Kumar et al., 2017).

### Statistical Analysis

All data was first checked for normality using the Kolmogorov–Smirnov test. To test our primary hypotheses and assess whether there was a drug effect on PAS-induced LTP, a repeated measures analysis of variance (rmANOVA) was conducted with the drug condition (placebo vs. baclofen, vs. dextromethorphan vs. L-DOPA vs. rivastigmine) as the repeated measure. The repeated measure factor that was used in the rmANOVA was the drug condition because each participant had PAS repeated five times, each time under one of the five drug conditions. rmANOVA was followed by a series of *post hoc* analyses, with Bonferroni correction, to compare PAS-induced LTP under each of the active drug conditions to PAS-induced LTP under placebo. Bonferroni correction was applied to the *post hoc* comparisons of each of the four active drugs to placebo, i.e., for four comparisons. In other words, the α-value for each comparison of a certain active drug to placebo was 0.0125.

To assess whether there was PAS-induced LTP under each drug condition, we ran a series of one-sample *t-*tests to compare PAS-induced LTP under each drug condition to a test value of 1 representing no LTP, a Bonferroni correction was also applied in this analysis.

In addition to the above primary analyses which are focused on the DLPFC electrode, i.e., the electrode at the site of stimulation, we divided all electrodes into six regions: Left Frontal (F1, F3, F5, F7, AF3, FP1, Right Frontal (F2, F4, F6, F8, AF4, FP2), Central (FPZ, FZ, FCZ, CZ, CPZ), Left Lateral (FC1, C1, CP1, FC3, C3, CP3, FC5, C5, CP5, FT7, T7, TP7), Right Lateral (FC2, 2, CP2, FC4, C4, CP4, FC6, C6, CP6, FT8, T8, TP8) and Posterior (PZ, P1-P8, POZ, PO3-PO8, OZ, O1, O2). We then averaged PAS-induced LTP across all electrodes within each region and conducted similar rmANOVAs to what is described above to assess the impact of each drug compared to placebo on PAS-induced LTP within each region as whole. The significance of these rmANOVAs were Bonferroni corrected by a factor of six, i.e., α-value for significance was 0.008.

## Results

Thirteen participants (4 females and 9 males) took part in this study. All participants completed all sessions except for one participant who dropped out after only one out of the five sessions and data for this participant was not used. Participants’ demographics and basic neurophysiological characteristics are described in Table 1.

**Table 1:** Demographic and basic neurophysiologic characteristics.

Characteristic	Mean (SD)
Age (years)	31.3 (10.5)
Gender (Female, %)	4 (25)
Education (years)	15.3 (2.3)
Resting motor threshold	49.0 (4.9)
SI1 mV	61.5 (8.3)
Peripheral nerve stimulation count*
Placebo	175.5 (9.6)
Baclofen	171.6 (11.2)
Dextromethorphan	183.3 (22.3)
L-DOPA	176.7 (6.0)
Rivastigmine	174.3 (7.1)
Pre-PAS CEA*
Placebo	895.88 (405.43)
Baclofen	1404.88 (2313.92)
Dextromethorphan	797.96 (446.30)
L-DOPA	814.04 (390.12)
Rivastigmine	796.71 (500.40)

*There was no significant drug effect on peripheral nerve stimulation count (*F*_(1.65,18.15)_ = 1.28, *p* = 0.30) or pre-PAS CEA (*F*(1.09,9.810) = 0.86, *p* = 0.39). For peripheral nerve stimulation, under each drug condition, the count did not differ significantly from the actual number of peripheral nerve stimulations (i.e., 180) (*p*’s > 0.05). SI 1 mV, Stimulation intensity with a mean peak-to-peak motor evoked potential amplitude of 1 mV over 20 trials; SD, standard deviation.

The electrode that was used each participant as site of stimulation and for the primary analyses were either F5 or F7 except for one session of one participant in which F3 was used.

All outcome data was normally distributed, rmANOVA revealed that there was a significant drug effect on PAS-induced LTP as measured from the electrode over the site of stimulation (*F*_(4,44)_ = 10.08, *p* < 0.001). Further, *post hoc* pairwise comparisons against placebo (PAS-induced LTP = 1.25, SD = 0.14), with a Bonferroni correction, revealed that LTP was significantly increased after the intake of L-DOPA (PAS-induced LTP = 1.64, SD = 0.37, *p* = 0.004) or rivastigmine (PAS-induced LTP = 1.63, SD = 0.40, *p* = 0.009) and decreased after the intake of dextromethorphan (PAS-induced LTP = 0.95, SD = 0.19, *p* = 0.007). In contrast, there was no change after the intake of baclofen (PAS-induced LTP = 1.15, SD = 0.52, *p* = 0.54; Figures 2, 3).

Compared to a test value of 1 which represents no LTP, participants experienced PAS-induced LTP under placebo, L-DOPA and rivastigmine, but not under baclofen or dextromethorphan condition after a Bonferroni correction (Table 2).

**Table 2:** Potentiation over the dorsolateral prefrontal cortex (DLPFC) under each drug condition.

Drug	PAS-induced LTP (SD)	*t* (df)	*p*-value
Placebo	1.25 (0.14)	4.31 (11)	0.001
Baclofen	1.15 (0.52)	1.0 (11)	0.34
Dextromethorphan	0.95 (0.19)	−0.95 (11)	0.36
L-DOPA	1.64 (0.37)	6.0 (11)	<0.001
Rivastigmine	1.63 (0.40)	5.36 (11)	<0.001

*PAS-induced LTP, Paired Associative Stimulation (PAS)-induced Long-Term Potentiation (LTP)-like activity as measured by Cortical Evoked Activity (CEA) post-drug/CEA pre-drug at maximum LTP-like activity; SD, standard deviation; *t*(df), one sample t-test (degrees of freedom) with a test value of 1 which is equivalent to no LTP*.

Finally, the region-based rmANOVAs revealed that there was a significant drug effect on PAS-induced LTP in only the Left Frontal region (*F*_(4,44)_ = 23.59, *p* < 0.001). There was no effect in the Right Frontal (*F*_(4,44)_ = 2.76, *p* = 0.039), Left Lateral (*F*_(4,44)_ = 0.60, *p* = 0.66), Right Lateral (*F*_(4,44)_ = 0.35, *p* = 0.85), Central (*F*_(4,44)_ = 0.79, *p* = 0.54), or Posterior (*F*_(4,44)_ = 0.75, *p* = 0.57) region. *Post hoc* pairwise comparisons against placebo (PAS-induced LTP = 1.05, SD = 0.20), with a Bonferroni correction, revealed that LTP was significantly different (increased) only after the intake of L-DOPA (PAS-induced LTP = 2.18, SD = 0.70, *p* = 0.002) but not rivastigmine (PAS-induced LTP = 1.14, SD = 0.19, *p* = 1.0), dextromethorphan (PAS-induced LTP = 0.97, SD = 0.22, *p* = 1.0), or baclofen (PAS-induced LTP = 1.13, SD = 0.17, *p* = 1.0).

## Discussion

This study confirmed our hypotheses that L-DOPA and rivastigmine enhanced neuroplasticity in the DLPFC *in vivo* and that dextromethorphan blocked it. It did not confirm the fourth hypothesis that baclofen reduces DLPFC neuroplasticity when compared to placebo although under baclofen exposure participants did not experience significant potentiation compared to baseline. To our knowledge, this is the first study to assess the pharmacological modulation of DLPFC neuroplasticity in humans.

Our finding that L-DOPA enhanced DLPFC neuroplasticity is consistent with animal studies that reported enhanced LTP in the prefrontal cortex following dopaminergic intervention (Otani, 2003). Dopaminergic neurons project from the ventral tegmental area to the prefrontal cortex. These projections activate dopamine D_1_ receptors on prefrontal pyramidal neurons and facilitate NMDA receptor activity (Seamans et al., 2001; Wang and O’Donnell, 2001). L-DOPA is a dopamine precursor that is converted to dopamine, which activates these dopaminergic receptors (Okereke, 2002), resulting in enhanced LTP.

Our finding is also consistent with human studies that assessed dopaminergic modulation of PAS-induced LTP in the motor cortex measured through MEP activity (Kuo et al., 2008; Nitsche et al., 2009; Korchounov and Ziemann, 2011; Thirugnanasambandam et al., 2011; Kishore et al., 2014). In the motor cortex, L-DOPA increased the magnitude and duration of PAS-induced LTP (Kuo et al., 2008). This effect was not affected by sulpiride (Ross et al., 2006; Nitsche et al., 2009), a D_2_ receptor antagonist, underlining the role of D_1_ receptors in L-DOPA enhancement of PAS-induced LTP (Monte-Silva et al., 2009).

We also found that rivastigmine enhanced DLPFC neuroplasticity. Rivastigmine increases synaptic levels of acetylcholine by inhibiting acetylcholine-esterase, allowing for longer cholinergic receptors activation (Polinsky, 1998). In animal and human brain slice studies, cholinergic activity plays a pivotal role in LTP facilitation in the prefrontal cortex (Vidal and Changeux, 1993). Several studies have shown that cholinergic agonists enhance LTP (Blitzer et al., 1990; Bröcher et al., 1992). This effect on LTP is thought to be mediated by a transient reduction in inhibitory transmission, which in turn, lowers the threshold for NMDA receptor dependent LTP, presynaptic nicotinic receptors activation and an increase in calcium influx, as well as postsynaptic muscarinic receptors activation and related intracellular signaling pathways (Metherate and Ashe, 1993; Jerusalinsky et al., 1997; Letzkus et al., 2011; Teles-Grilo Ruivo and Mellor, 2013). In the human motor cortex, biperiden, a muscarinic M1 receptor cholinergic antagonist suppressed (Korchounov and Ziemann, 2011), while rivastigmine enhanced PAS-induced LTP measured through MEP (Kuo et al., 2007).

Our third and confirmed hypothesis was that dextromethorphan blocks DLPFC neuroplasticity. This finding is consistent with previous studies assessing the effects of dextromethorphan on LTP in animal and human studies (Krug, 1993; Stefan et al., 2002; Weise et al., 2017). Dextromethorphan is a non-competitive NMDA receptor antagonist (Church et al., 1985). Thus, it is expected to suppress NMDA-receptor dependent LTP. Our finding with dextromethorphan also provides evidence that PAS-induced LTP in the DLPFC represents synaptic LTP by being dependent on functional NMDA receptors similar to cellular LTP.

Contrary to our fourth hypothesis, we did not find a difference in PAS-induced LTP under baclofen compared to placebo. However, we still found that under baclofen exposure, participants did not experience significant PAS-induced LTP compared to baseline (pre-PAS). Our original hypothesis was based on a study that assessed the effects of baclofen in the motor cortex which included only five participants (McDonnell et al., 2007). Thus, the discrepancy may be due to difference in brain region. Baclofen is a GABA_B_ receptor agonist that can post-synaptically suppress PAS-induced LTP or enhance PAS-induced LTP via presynaptic GABA_B_ receptors, as it could also lead to decreased release of GABA through GABA_B_ receptor-mediated auto inhibition (Jablensky, 1997). For instance, it has been shown in mice that the deletion of GABA_B_ auto-receptors led to a failure in LTP expression (Vigot et al., 2006).

This study is limited by a relatively small sample size. However, the sample size was calculated based on previously published literature in the motor cortex. Another limitation is that we did not measure blood levels of the drugs prior to the delivery of PAS. However, this limitation is mitigated by administrating PAS based on published plasma peak values of the drugs. Further, this study assessed the impact of a single dose on PAS-induced LTP. These medications are used chronically in clinical settings. Thus, future studies should assess the effects of chronic exposure to these medications in healthy individuals as well as patients with brain disorders associated with abnormalities in these neurochemical systems. One more limitation is the fact that low frequency rTMS protocol can impact brain excitability. While this is a limitation of several TMS-EEG protocols, the placebo arm in our study design would mitigate the impact of this confound. Another limitation of the experimental design is that we administered the drug after pre-PAS rather than before pre-PAS, confounding the effect post-PAS by a direct effect on CEA and not necessarily through PAS. However, this confound is at least partially mitigated by the fact that, as detailed above, several studies showed no significant impact of these drugs on basic neurophysiological measures. Finally, we did not use auditory masking to control for auditory artifacts due to several reasons. First, given the length of these experiments, it would have been extremely uncomfortable for a participant to wear earplugs for the entire duration of the study. This would have, in turn, affected the participant’s state and interfered with the quality of data. Second, considering that this sound can travel through the air and the bone, the auditory evoked potentials cannot be fully obscured by wearing earplugs or using white noise (Nikouline et al., 1999). Third, our pre-PAS and post-PAS conditions were administered in the same manner, which should control for the auditory artifact. That is, any small auditory artifact is not anticipated to have influenced the effects of any one pharmacological agent on PAS more so than any other. Further, we also concatenated the EEG data for pre-PAS condition with post-PAS conditions. As such, no one involved in the study was aware of which condition was pre-PAS or post-PAS and all analyses were done under completely blind conditions, further enhancing the objectivity of data cleaning. Lastly, our work assessing cortical inhibition using TMS-EEG demonstrated that CEA in the DLPFC was unaffected when the auditory artifact was subtracted out using a sham experiment while the suppression of CEA remained significant (Farzan et al., 2009).

In conclusion, this is the first study to investigate the pharmacological modulation of DLPFC neuroplasticity in humans. The study confirmed our hypotheses that dopaminergic and cholinergic neurotransmission enhance DLPFC neuroplasticity while suppressing glutamatergic neurotransmission blocks it. Future studies could assess a time dynamic analysis of these pharmacological modulations of neuroplasticity using a larger sample size of healthy individuals. Other future studies should also assess these modulations in clinical conditions to better understand the pathophysiology underlying these conditions as well the mechanisms that these drugs target in various brain disorders.

## Author Contributions

BS, ZJD and TKR first conceptualized and designed the study. BGP, DMB and RC contributed to the conceptualization. BS and TKR wrote the first main draft of the manuscript. BS, ZJD and TKR revised and prepared the final draft. All authors revised and edited the final version. BS prepared Figure 1. BS and RZ prepared Figures 2, 3. RZ prepared Figure 4.

## Conflict of Interest Statement

The authors declare that the research was conducted in the absence of any commercial or financial relationships that could be construed as a potential conflict of interest.
